# Distribution of human papillomaviruses and bacterial vaginosis in HIV positive women with abnormal cytology in Mombasa, Kenya

**DOI:** 10.1186/s13027-016-0061-1

**Published:** 2016-04-06

**Authors:** Sonia Simone Menon, Rodolfo Rossi, Ronald Harebottle, Hillary Mabeya, Davy vanden Broeck

**Affiliations:** International Centre for Reproductive Health (ICRH), Ghent University, De Pintelaan 185 P3, 9000 Ghent, Belgium; Ambior Research group, Belgium (LSHTM Alumni), Ghent, Belgium; Horizon Health Network, New Brunswick, Canada; Moi University/Gynocare Fistula Centre, Eldoret, Uasin Gishu Kenya; Faculty of Medicine and Health Sciences, AMBIOR (Applied Molecular Biology Research Group), Laboratory of Cell Biology & Histology, University of Antwerpen, Antwerpen, Belgium

**Keywords:** HIV, HPV, CD4 count, BV, Cervicitis

## Abstract

**Background:**

HPV is the major etiological factor in the causal pathway for cervical cancer, which is the leading cancer among women in sub-Saharan Africa. HIV is associated with a higher prevalence and a broader range of high-risk HPV genotypes. Studies have shown a positive association between Bacterial vaginosis (BV) and HPV and HIV. Also, in African women, BV was found to be significantly associated with vaginal inflammation. The high prevalence of BV, HIV and HPV infections in the African continent makes elucidation of the interactions with one another of utmost public health interest. The aims of the current study are to examine the frequency of HPV genotypes and BV as well as their respective risk factors within an HIV infected population with abnormal cytology in the resource-constrained setting of Mombasa, Kenya and, secondly, highlight issues to consider for triple co-infection clinical management.

**Method:**

Cross-sectional analysis with a sample drawn from an ongoing cohort study. All consenting, non-pregnant HIV infected women, between 18 and 50 years of age, without a history of cervical cancer or hysterectomy, between November 2005 and April 2006 were screened for HR HPV DNA in Mombasa, Kenya. 1 out of 4 HIV positive women fulfilled the criteria by having SIL (24.9 %). 600 HIV infected women were tested to reach a cohort of 74 HIV women with abnormal cytology. To assess which factors were associated with HR HPV, crude statistical analysis was performed through logistic regression.

**Results:**

Bacterial vaginosis (BV) was found in 46 women out of 74 (62.2 %). Cervicitis was diagnosed in 15 % of women (*n* = 11), of which 8 had BV. The most prevalent HPV genotypes were HPV 16 (33.8), HPV 53 (24.3) and HPV 18 (17.6 %), while 65 % of the participants had multiple genotype infection.

Statistically significant associations between CD4 counts <200 cells/μl and multiple HPV prevalence, adjusted for age were also noted (OR = 3.7; 95 CI: 1.2–12.1; *p* = 0.03) and HPV53 (OR = 4.4, 95 % CI: 1.4–13.6; *p* = 0.01). A statistically significant association was found between CD4 count ≥ 350 μl and HPV 16 adjusted for age (OR = 2.9; 95 % CI: 1.0- 8.3; *p* = 0.05). A borderline statistically significant association was observed between BV and HPV58 (crude OR = 4.1, 95 % CI: 0.8–21.0; *p* = 0.07).

**Conclusion:**

The most prevalent HPV genotypes observed were HPV 16, HPV 53, and HPV 18, which have a combined prevalence of 76 %. Our results show that a triage based on CD4 count should start at CD4 count ≥ 350 μl as our study suggests that HPV 16 are more prevalent when women are moderately immunosuppressed. Given the high prevalence of HPV 53 in a HIV infected population with abnormal cytology, its cervical carcinoma genesis potential as a stand-alone genotype and as well as its synergism with multiple infections should be investigated. The new WHO guideline in resource-poor settings to rescreen women for HPV within ten years may be more effective if BV and cervicitis management become a major component for HIV-HPV management.

## Background

Cervical carcinoma is the fourth most prevalent cancer in the world and the most common female cancer in sub-Saharan Africa [1]. It is the second most prevalent cancer among women in Kenya, after breast cancer, and its incidence is increasing [2].

Infection with a high risk (HR) Human Papillomavirus (HPV), a sexually transmitted DNA virus, is the central etiological agent in the development of cervical cancer and include HPV genotypes 16, 18, 31, 33, 35, 39, 45, 51, 52, 56, 58, 59 and 68 [3]. Others HPV types 26, 53, 66, 67, 70, 73, and 82 are classified as “possible/probable” (pHR) carcinogens according to the recent review of International Agency for Research on Cancer (IARC) assessing carcinogenicity of biological agents [4]. The 15 HR oncological viral strains, which have been identified can be broken down into the HPV 16 group (alpha-9) of the alpha-papillomavirus genus (HPV 31, HPV33, HPV 35, HPV 52, and HPV 58) and the HPV 18 group (alpha-7; HPV39, HPV 45, HPV 59, and HPV 68) [5, 6].

Cervical cancer is the result of a change in the cell cycle control caused by HP. Cervical intraepithelial neoplasia (CIN) can be histologically graded into mild dysplasia (CIN 1), moderate dysplasia (CIN 2), and both severe dysplasia and carcinoma in situ belonging to CIN 3 [7].

Apart from a higher prevalence and broader range of HR HPV, HIV immunosuppression has been linked to multiple HPV infection [8, 9], particularly in those with CD4 count <200 cells/μl [10], meaning when immunosuppression becomes severe. Multiple HPV genotypes co-infections has been attributed to the inability to clear HPV infections as well as to the reactivation of latent HPV infections, both occurring as a result of immune suppression [6, 8, 11, 12]. In a recent large study on multiple HPV infections in Costa Rica, young non-HIV positive women with multiple infections were observed to be at significantly increased risk of CIN 2+, when compared with those with single infections [13, 14].

Moreover, concurrent genital infections are common in HIV-1–infected women. The prevalence of Bacterial Vaginosis (BV), characterized by an overgrowth of vaginal anaerobic flora and reduction of H_2_0_2_-producing lactobacilli in African women is among the highest worldwide [15], which is of particular concern, as there is evidence that BV is a risk factor for acquisition and transmission of many STI’s, including HIV and HPV [16, 17]. A recent meta-analysis of available literature also suggested a positive association between BV and HPV infection, though no study in Africa was eligible to be included [18]. A recent HIV Epidemiology Research study in Tanzania showed that BV is also associated with increased odds for incident HPV as well as delayed clearance among women [19]. These findings are in line with biologic plausibility since, unlike most cervical HPV infections, BV causes major changes in the local vaginal environment leading to degradation of innate defenses.

In previous studies, BV was associated with many sexually transmitted infections (STIs), including infection with *Chlamydia trachomatis*, *Neisseria gonorrhoea*, HSV-1 and 2 [20–22]. Also, there is evidence that BV is an independent predictor for HPV 16 and its related genotypes [23]. There is growing evidence from other studies that BV is an independent risk factor for cervicitis [24]. BV could lead to cervicitis through a loss of bactericidal H_2_0_2_-producing lactobacilli, reduced levels of protective vaginal mucins, and increased pro-inflammatory enzymes and cytokines, which in turn may decrease the cervical mucus barrier [24].

New guidelines from WHO in December 2014 advocates for a “screen and treat” approach in resource poor settings, in which treatment of detected precancerous lesions ensues Visual Inspection with Acetic acid (VIAC) or HPV testing for screening soon or immediately [25]. The use of HPV tests for cervical cancer prevention is expected to reduce the frequency of screening and “once a woman has been screened negative, she should not be rescreened for at least 5 years, but should be rescreened within ten” [23]. However, there is current scant evidence to support the adequateness of this guideline in HIV-infected women living in low resource settings. Screening programs need to be tailored to the resources and capacity in each area. This secondary preventive strategy would accompany the comprehensive strategy to prevent cervical cancer in Kenya, which includes plans for vaccinating girls with the quadrivalent vaccine (Gardasil™) by 2015 [26, 27].

This analysis of HIV-positive women with abnormal cytology purports to test our a priori hypotheses that HPV 53, being more prevalent in HIV-infected women is associated with immunosuppression and that BV is associated with genotypes pertaining to the alpha-group. The objectives are threefold: 1) to describe the distribution of HPV genotypes and multiple HPV infections, cervicitis, BV and STIs within our population; 2) to explore whether low CD4 count is a predictor for multiple HR HPV genotypes and the most prevalent pHR/HR HPV genotypes and BV a predictor for alpha 9 related genotypes as well as the most prevalent pHR/HR HPV genotypes 3) discuss the potential effectiveness of a screening protocol triaged by CD4 count and age and the public heath management of HIV-HPV-BV.

## Methods

This cross-sectional analysis reports the findings of a sample of women enrolled in a prospective cohort study, whose aim was to define the best way to manage low grade squamous intraepithelial lesions in HIV-1 infected women, aged between 18 and 50 years in Mombasa, Kenya, between November 2005 and April 2006. As all women were to receive the same intervention depending on their squamous intraepithelial lesion, no formal sample size calculation was needed. 600 women were screened within this period. Non-pregnant women of reproductive age and attending the Comprehensive Care Centre for management of HIV infection at Coast Province General Hospital in Mombasa, Kenya, were invited to participate. Within the 600 HIV positive women undergoing cytological examination, we expected to find 10 % prevalence of Squamous Intraepithelial Lesions (SIL) for the HIV positive patients not on HAART and 15 % for those under HAART period, resulting in 74 HIV women with abnormal cytology being recruited from the initial cohort.

Cytological abnormalities, a prerequisite for participating in the study, were all histologically confirmed. Exclusion criteria included pregnancy, less than 6 weeks post-partum, history of cervical cancer or hysterectomy. Self-reported behavioral risk factor assessment included the presence of an STI, number of sexual partners, the age of first sexual intercourse and regular use of a condom. Blood plasma samples for measuring CD4 count were taken, and a gynaecological examination was performed with speculum insertion, and collection of endocervical and high vaginal swabs.

### Biologic specimens

Cervical samples were collected using a cervix brush (Cervex-brush®, Rovers®, Oss, The Netherlands), and cervical cytology was assessed with conventional Papanicolaou (Pap) smears. Slides were read by a cytologist with master level training, supervised by a pathologist. An external cytopathologist provided quality control. The Bethesda Reporting System was used for cytological classification [28].

The cervix brush tips were preserved in a liquid-based cytology collection medium (SurePath®, Tripath Imaging Inc., Burlington, North Carolina, USA) and stored at 4 °C until further processing.

### HPV DNA extraction, detection and typing

HPV testing was done as described by Depuydt et al. (2006) Micalessi IM et al. (2012) in an accredited laboratory (ISO certification: ISO15189) [29, 30]. Briefly, HPV DNA was extracted from exfoliated cervical cells using the standard proteinase K-based digestion protocol, following the manufacturer’s instructions. Cells were incubated with proteinase K solution (100 μg/ml) for 3 h at 55 °C. DNA was then further purified by spin column chromatography. HPV types were determined using a series of real-time PCR reactions with specific primers and TaqMan® (Invitrogen, La Jolla, USA) probes for HR HPV types 16, 18, 31, 33, 35, 39, 45, 51, 52, 53, 56, 58, 59, 66 and 68 IARC (2006). Low risk (LR) HPV types 6 and 67 were also detected. HPV DNA was tested according to de Meijers’ et al. (2009) guidelines for HPV DNA test requirements [31].

The national recommendation was followed to diagnose HIV and a parallel testing algorithm was used for HIV diagnosis using rapid immunoassays: Uni-Gold™ Recombigen® HIV (Trinity Biotech plc, Bray, Ireland) and Determine® HIV-1/2 (Abbott Japan co Ltd, Minato-Ku, Tokyo, Japan). In the case of indeterminate results, an enzyme-linked immunosorbent assay was used to confirm HIV status.

### Diagnosis of STIs

A gynecological examination was performed and blood, urine, PAP smear, and high vaginal and endocervical swabs collected. PAP smears were read using conventional cytology and classified using the Bethesda classification. Infection with *Trichomonas vaginalis* was determined by wet mount and/or PAP smear slides. To test for *Neisseria gonorrhoeae* infection, culture plates (blood agar; International Diagnostic Group, Lancashire United Kingdom) were inoculated with endocervical swabs Presence of HIV was confirmed in the patient’s serum using a parallel algorithm comprising Uni-Gold® HIV (Trinity Biotech plc, Bray, Ireland) and Determine® HIV-1/2 (Abbott Japan co Ltd, Minato-Ku, Tokyo, Japan). Elisa was used when parallel test results were discordant.

### Cervicitis

Cervicitis was diagnosed from inflammatory cells by means of a microscopic test.

### Statistical analysis

Analysis was done using STATA version 12 (Stata-Corp LP, College Station, TX).

Age was dichotomized into > 30 years and ≤ 30 years; this categorization was used to reflect the WHO 2014 guideline concerning cervical screening. Condom use was dichotomized as always or irregular, according to self-report. CD4 cell count was analyzed both as a continuous variable and as two categorical variable, using a cut off of CD4 count <200 cells/μl (vs ≥ 200 cells/μl) and CD4 count <350 cells/μl (vs ≥350 cells/μl), when HIV infected women are severely immunosuppressed and moderately immunosuppressed, respectively. The number of sexual partners was also dichotomized as per other studies (up to 5 versus 6 or more), this dichotomization was used to make our studies more comparable to others and to explore the risk from a public health point of view.

The method employed for the first objective was to calculate the proportion of the specific HPV genotype divided by 74 (the sample size), the number of co-infected women with abnormal cytology, as well as the proportion of STI, BV and cervicitis, along with a 95 % CI. For the second objective, logistic regression analysis was performed to identify risk factors for HR-HPV. This included the associations between low CD4 count and the most prevalent pHR/HR HPV genotypes (as well as the one between BV and alpha 9 phylogenetically-related genotypes and most prevalent pHR/HR HPV genotypes. This outcome variable was dichotomized into low-risk HPV (LR-HPV) and absence of HPV genotypes versus HR-HPV genotypes (see above). The resulting odds ratio was used to measure the strength of the association between HPV infection and each risk factor in turn. A multivariable logistic regression analysis was performed to simultaneously control for potential confounders and to assess the adjusted association for various risk factors. To assess the impact of CD4 count on the effects of the parameters, interaction parameters were assessed in the models by doing a Wald test. As no interaction terms were significant, no model reflecting the differential impact for that particular outcome was included.

The same method was also used to explore the simultaneous effect of age and number of sexual partners on the HPV genotype and to explore whether there was an association between the two or more HR HPV co-infections and CD4 count, while adjusting for age and the number of sexual partners.

Statistical tests were considered significant when the *p*-value, derived from the Wald Test, is 0.05 or less.

### Ethical considerations

Staff obtained written informed consent from patients, collected demographic and behavioural data using structured questionnaires. All human subject protocols were approved by the Ethics Committee at the Kenyatta National Hospital, which also gave overall ethical approval for this study (Ref: KNH-ERC/01/3618).

## Results

### Characteristics of the population

The study included 74 non-pregnant HIV-infected women with an abnormal cytology. The mean age was 34.2 years of age (SD = 6.5). The median age at first sexual intercourse was 18.0 (IQR: 15.5–20.0). The median number of sex partners was 2 (IQR: 1–4). The median CD4 count was 236 cells μl/(IQR: 158–374). The mean number of HR HPV genotypes per participant was 2.3 (SD = 1.6). The majority of patients (82 %) were under treatment with highly active antiretroviral therapy (HAART) (Table 1). Concerning sexual behavior, 81.8 of women reported irregular use of condoms, and 87.9 % had up to 5 sexual partners (Table 1).

**Table 1:** Distribution of various categorical variables: age, sexual behaviour and CD4 count

Variable	*N*	Percentage (95 % CI)
Age group:		
> 30 years	54	73.0 % (61.4–82.6)
≤ 30 years	20	27.0 % (17.4–38.6)
Sexual behaviour:		
First sexual encounter <15 years old	29	60.4 % (52.7–74.2)
First sexual encounter > =15 years old	19	39.6 % (25.8–54.7)
> 6 sexual partners	7	12.1 % (5.0–23.3)
≤ 5 sexual partners	51	87.9 % (76.7–95.0)
Regular use of condom	10	18.2 % (9.1–30.9)
No regular use of condom	45	81.8 % (71.3–92.3)
CD4 count:		
CD4 count <200 cells/μl	26	35.1 % (24.0–46.3)
CD4 count ≥200 cells/μl	48	64.9 % (69.1–91.0)
CD4 count <350 cells/μl	52	70.3 % (58.5–80.3)
CD4 count ≥350 cells/μl	22	29.7 % (19.1–40.4)

### Prevalence of cervical abnormalities

Atypical squamous cells with possible high significance (ASC-H) were detected in 4.0 %, atypical squamous cells of undetermined significance (ASC-US) in 16.2, LSIL 58.1 and HSIL in 20.3 % of the patients.

Histology results found the following abnormalities: Cervical intraepithelial neoplasia (CIN I) in 58.3 %, CIN 2+ 43.2 %, of which 1 had ICC. Cervicitis was recorded in 15 and normal biopsy in 4.0 % of the participants. In 5 women with CIN 1, HPV was absent and in the two normal biopsies, HPV was present.

### Prevalence of HPV genotypes

The most prevalent HPV genotypes found in this HIV-infected cohort of women are displayed in Table 1. HPV 16 and 53 were the most prevalent, with 34.8 and 24.3 %, respectively followed by HPV 18 with 17.6 % (Table 2).

**Table 2:** Prevalence of pHR/HR and low risk HPV infection and other sexually transmitted infections

Type of infection	*N*	Percentage (95 % CI)
HR HPV:		
HPV 16	25	33.8 % (22.8–44.8)
HPV 18	13	17.6 % (8.7–26.4)
HPV 31	11	14.9 % (6.6–23.2)
HPV 33	12	16.2 % (7.6–24.8)
HPV 35	13	17.6 % (8.7–26.4)
HPV 45	3	4.1 % (0.5–8.7)
HPV 39	8	10.8 % (3.6–18.1)
HPV 51	11	14.9 % (6.6–23.2)
HPV 52	15	20.3 % (10.9–29.6)
HPV 53	18	24.3 % (14.3–34.4)
HPV 56	15	20.3 % (10.9–29.6)
HPV 58	13	17.6 % (8.7–26.4)
HPV 66	11	14.9 % (6.6–23.2)
HPV 68	4	5.4 % (0.1–10.7)
Multiple HR HPV	48	64.9 % (53.7–76.0)
Women on HAART	60	82.0 (80.0–90.2)
LR HPV:		
HPV 6	4	5.4 % (4.4–6.4)
HPV 67	0	0
No HPV infection:	6	8.1 % (3.0–16.8)
STIs:		
BV	46	62.2 % (50.9–73.5)
Genital ulcer	12	16.2 % (7.6–24.8)
Genital warts	8	10.8 % (3.6–18.1)
Trichomonas vaginalis	1	1.4 % (0.1–4.0)
Cervicitis	11	14.9 % (6.6–23.2)
STI prevalence	51	68.9 % (58.1–79.7)
More than one STI	8	10.7 % (3.5–17.8)

A large proportion, 64.9 %, of the women in this cohort has multiple HPV genotypes (Table 2). Most multiple infections were dual infections, (24 %), but two women had up to 7 co-infections (Table 3).

**Table 3:** Prevalence of multiple HPV genotypes among the total sample size (*N* = 74)

Number of co-infections	*n.*	%
2 co-infections	18	24 %
3 co-infections	12	16 %
4 co-infections	12	16 %
5 co-infections	3	4 %
6 co-infections	1	1 %
7 co-infections	2	3 %
Total	48	65 %

At least one of the HR HPV genotypes was observed in 86.5 of the women, 5.4 with LR-HPV types and 8.1 % without any contemporaneous HPV infection.

### Prevalence of concurrent STIs

STIs were observed in 68.9 of all women, and 15.7 % had more than 1 STI co-infection (Table 1). 16.2 were diagnosed with genital ulcer disease, 1.4 *trichomonas vaginalis* and 10.8 % had genital warts.

### Sexually enhanced disease

Out of the 11 women with cervicitis, 8 had BV.

### Risk factors for specific HR-HPV infection: results of uni- and multivariable analysis

No significant association was observed between HPV 16 and age (OR = 1.4; 95 % CI: 0.5–3.6; *p* = 0.5), adjusted for ≥6 sexual partners (aOR = 1.1; 95 % CI: 0.3–4.1; *p* = 0.8); HPV 53: (OR = 05; 95 % CI: 0.2–1.6; *p* = 0.3) adjusted for ≥6 partners (aOR = 0.9; 95 % CI: 0.2–3.2; *p* = 0.8) and HPV 18 (OR = 0.4; 95 % CI 0.1–1.6; *p* = 0.2): adjusted for ≥6 partners (OR = 2.3; 95 % CI: 0.5–11.5; *p* = 0.3). No significant association was found for multiple HPV genotypes and age (OR = 1.8; 95 % CI: 0.6–5.1; *p* = 0.3) and adjusted for sexual partners (OR = 2.4; 95 % CI: 0.7–8.9; *p* = 0.2) (Table 4).

**Table 4:** Age-adjusted association between specific pHR/HR HPV genotypes and CD4 count < 200 μl and CD4 count ≥350 cells/μl (upper part); sex -adjusted associations between the three most prevalent pHR and HR HPV genotypes and age OR from logistic regression

HPV genotype	Odds ratio	95 % CI	*P* value*
CD4 count < 200 cells/μl			
HPV 16	0.7	0.2–1.9	0.4
HPV 18	1.3	0.4–4.6	0.7
HPV 31	1.1	0.3–4.4	0.9
HPV 33	3.3	0.9–11.9	0.07
HPV 35	2.4	0.7–8.3	0.2
HPV 45	3.3	0.3–39.4	0.4
HPV 51	1.5	0.4–5.6	0.6
HPV 52	2.5	0.8 8.1	0.1
HPV 53	4.4	1.4–13.6	0.01
HPV 56	0.6	0.2–2.2	0.4
HPV 58	2.6	0.7–8.7	0.1
HPV 66	3.1	0.8–11.9	0.1
HPV 68	0.6	0.06–6.2	0.7
Multiple HPV co-infection	3.7	1.2–12.1	0.03
CD4 count ≥350 cells/μl			
HPV 16	2.9	1.0–8.3	0.05
HPV 18	0.4	0.08–1.9	0.2
HPV 53	0.6	0.2–2.1	0.4
Multiple HPV co-infection	0.7	0.3–2.0	0.5
Age ≥ 30 adjusted for sexual partners			
HPV 16	1.1	0.3–4.1	0.8
HPV 18	2.3	0.5–11.5	0.3
HPV 53	0.9	0.2–3.2	0.8
Multiple pHR/HR HPV infections	2.4	0.7–8.9	0.2

*p-value from Wald test

### CD4 count

35.1 % women had low CD4 counts <200 cells/μl and 64.9 % had high CD4 count ≥200 cells/μl. 70.3 had CD4 count <350 cells/μl and 29.7 % had CD4 count ≥350 cells/μl (Table 3). The OR obtained from the logistic regression yielded a statistically significant association between CD4 < 200 μl and multiple HPV co-infections (OR: 3.7; 95 % CI: 1.2–12.1; *p* = 0.03) but a non-significant association between CD4 count ≥350 cells/μl and multiple HPV infections (OR: 0.7; 95 % CI: 0.3–2.0; *p* = 0.5). Low CD4 counts <200 cells/μl was found to be a significant predictor of only HPV 53 (age-adjusted OR = 4.4, 95 % CI: 1.4–13.6; *p* = 0.01) and HPV 16 (age-adjusted OR = 2.9; 95 % CI: 1.0–8.3; *p* = 0.05) (Table 4).

A *t*-test indicated that women with CD4 count <200 cells/μl have a significantly higher mean of prevalent HPV infections (mean of pHR HPV genotypes coinfections = 3.03) compared to women with CD4 count >200 (mean of pHR HPV genotypes coinfections = 2.0) : (*p* = 0.006 for the difference in mean). However, there was no significant difference (*p* = 0.5) between women with a CD4 count <350 cells/μl (mean = 2.4) in and women with CD4 count ≥350 cells/μl (mean = 2.1).

### Micro-organisms

68.9 had a concomitant STI, and 62.2 % women had BV (Table 1). With the exception of HPV 58 (OR = 4.1; 95 % CI: 0.8–21.0, *p* = 0.07), no significant association was found between BV and HPV 16 and its phylogenetically-related genotypes, HPV 31, HPV 33, HPV 58, HPV 52 (Table 5).

**Table 5:** Crude association between specific HR HPV genotypes and Bacterial vaginosis

HR HPV	OR^a^	95 % CI	*P* value*
Multiple pHR/HR HPV genotypes	1.0	0.4–2.8	0.9
HPV 16	0.5	0.2–1.4	0.2
HPV 18	1.0	0.3–3.3	1
HPV 52	0.6	0.2–2.0	0.4
HPV 53	0.9	0.3–2.8	0.9
HPV 58	4.1	0.8–20.0	0.07

^a^OR from Logistic regression

*P-value from Wald Test

## Discussion

### Summary of results and comparison with other studies

Our analysis shows that the most common pHR/HR HPV genotypes, HPV 16, followed by HPV 53 and HPV 18, with a combined prevalence of 76 %, were found in presence of other pHR/HR HPV infections. Strong associations were observed between HPV 53 and multiple HR HPV infections with CD4 count <200 cells/μl.

In agreement with studies showing HPV 16 to be the least affected by diminished immunity [12], we found that women with CD4 < 200 cells/μl had a 26.9 % prevalence of HPV 16 compared to 37.5 % in women with CD4 count >200/μl and a statically significant association between women with CD4 count ≥350/μl when adjusted for age. One study in Kenya found a significantly higher prevalence of HPV 16 in an HIV clinic where the median CD4 count at recruitment was 407 cells/μl [32]. Other studies in Kenya lacked stratification of HPV 16 and HPV 18 by HIV status and CD4 count. In a family planning unselected study conducted in Nairobi, it was observed that less than 10 % had HPV 16 and/or 18 these types were present in about 40 % of HSIL lesions [33]. In a study on FSW in Kenya, HIV infected women with normal cytology had an HPV 16 or 18 prevalence of 9.6 % [33], though the prevalence of either HPV is unknown in HIV infected women with abnormal cytology [34]. A hypothesis is that HPV16 may be more sensitive to attack from other genotypes, and thus may be at higher risk of competition when there is more immune suppression.

In our study, we detected a marginal association between BV and HPV 58, a HPV 16- related types, which is line with a study that detected a significant association between BV and HPV16-related types [23]. The fact that we only found a marginal significance is likely to be due to the sample size.

In our study a HPV 18 prevalence of 17.6 was observed, of which 54 had CIN 1 and 46 % had CIN2+. Our high HPV 18 prevalence was significantly higher than the 10.6 % observed by de Vuyst et al. in Kenya. Women with CD4 < 200 cells/μl appeared to have 20 % higher odds of being infected with HPV 18, although these associations were not statistically significant, (OR = 1.2; 95 % CI: 0.3–4.1). However, a South African study suggested that the prevalence of HPV 18 is inversely correlated with the level of immunosuppression, (10.6 %) in women with CIN 2/3 and 5 % with women with normal cytology [26].

A high prevalence of HPV53 had also been observed in other studies from Kenya. A study in HIV positive women (median CD4 count 281/μl) from Nairobi found high numbers of HPV53 in normal smears, whilst rare in HIV negative women [35]. In a Nairobi cohort with a median CD4 count of 538 cells/μl, HPV53 was among the most common HPV type (28.5 %) [36].

Although, intuitively, it would seem reasonable that all HPV genotypes should increase in frequency in HIV positive individuals, strong statistically significant associations were only detected between CD4 count <200 cells/μl and HPV53, the second most prevalent potentially oncogenic HPV genotype in our study (aOR = 4.4; *p* = 0.01; 95 % CI: 1.4–13.6) and the prevalence of multiple infection. Cervical HPV infection with multiple genotypes in HIV infected women has already been reported in regional studies [37–40].

Whilst younger age has been linked with higher prevalence of HR-HPV genotypes in HIV-negative women [41], we did not observe any statistically significant association between multiple HR HPV genotypes and age, in agreement with Luchters et al. [42]. One plausible explanation given was that the absence of age effects among HIV-infected women may have been due to a decreased ability to clear HPV infection, reactivation of HPV or re-infection with HPV types [12] previously cleared, all of which likely occur more commonly in HIV-infected women.

Estimates in BV range from 20 % to 50 % in African populations [43, 44]. A recent longitudinal study conducted in Kenya, Rwanda and South-Africa in HIV-negative women [45] reported a prevalence of 38 % [45]. The higher prevalence of BV (62 %) found in our study can be attributed to higher risk that women with BV face of contracting both HIV and HPV. In our study we found that 10.7 % of women with BV harboured other STIs. Inflammation related to BV is of concern because BV prevalence is very high in this population. On bivariate analysis, the association between BV and cervicitis failed to reach significance, but this may reflect insufficient power due to the number of BV-affected patients enrolled.

### Limitations and strengths of the study

A major strength of our study is that our analysis is limited to histological, considered to be the gold standard instead of cytological endpoints. However, the total sample size was rather limited, which may have occulted some plausible association of risk factors which may be evident with a larger sample size; for this reason, adjustment for various factors was limited. In addition, another limitation relates to the cross-sectional design, where the simultaneous data collection of BV and acquisition of HIV infection, as well as all other variables, the temporal criterion of causality is not fulfilled, thereby reversal causality cannot be excluded. These limitations may result in a sub-optimal internal validity of our study.

Our results can be generalized to other women receiving HIV care management. Our lack of association found between age and the most common HPV genotypes and multiple HPV genotypes do not appear to support the recommendation for a screen and treat protocol for women starting from thirty years of age. Also, a CD4 based triage may not be effective as HR HPV genotypes are seen at different levels of immunosuppression, at both CD4 < 200 cells/μl and CD4 count ≥350 cells/μl.

The new WHO 2014 HPV screen and treat recommendation comes with close timing with the WHO 2013 recommendation to initiate HAART when asymptomatic at CD4 count ≥350 cells/μl, which may offer opportunities to prevent HPV 53 and multiple HPV infections, thereby potential cervical dysplasia although it may not prevent HPV 16 and HPV 18. However, given the limitation of this study, including small sample size and its cross-sectional design, a prospective cohort or RCT with a larger size is warranted to elucidate potential relevance to plan screening according to the level of immunosuppression.

Biologic susceptibility to HPV acquisition and immune competence for clearance of an HPV infection could be affected by BV underscores the importance of prevention and successful treatment of BV. In light of this finding, the new WHO guideline to rescreen within ten years may be more effective if it is implemented in conjunction with a BV screening programme. Given the high prevalence of cervicitis in this population and its potential public health impact, it is important that BV clinical management becomes a major component for HIV-HPV management. To this effect, more health care personnel, including front line personnel, including nurses who make up the majority of the work force in sub Saharan Africa should be trained in microscope, Nugent score or Amsel reading of BV in HIV positive women. Furthermore, symptomatic management algorithm be established for cervicitis in this study population and its aetiology elucidated.

Effective treatment of cervicitis resulting in significant decreases in shedding of HIV-1 virus and infected cells in cervical secretions depends on the aetiology of BV and other pathogens involved implicated in cervicitis. In this study, cervicitis was common and predominantly non-gonococcal, although it is not possible to say that it was non-chlamydial in etiology as CT was not diagnosed and was found predominantly in women with BV.

Although high prevalence cervicitis has been reported in Africa, local epidemiological data regarding the prevalence, etiologies, and risk factors for cervicitis in HIV-HPV co-infected women in Kenya is lacking for guiding syndromic management of cervicitis in this group. The high prevalence in this study group may also affect sexual health at a population level as inflammation in the genital tract results in shedding of HIV in HIV-positive women may increase HIV to the general population. A recent meta analysis including HIV viral load observations in blood plasma, showed the average effect of a STI co-infection on HIV viral load in individuals on HAART was unlikely to decrease the effectiveness of treatment as prevention, although there is evidence of HIV compartmentalization in some treated patients where viral loads as measured in genital secretions are congruent with a non-negligible risk of transmission despite very low blood plasma viral loads [46–48].

Our marginal association between HPV 16 genetically related HPV 58 as well as the high prevalence of BV in HIV-HPV co-infected women along with a high prevalence of STIs underscores the need to elucidate its synergistic effects in increasing risk of HIV and HPV acquisition. Finally, the high level of co-infection with STIs underscores the value of screening for the simultaneous presence of different genital infections in HPV positive patients. This study also evidenced the lack of protective precautions against STIs, notwithstanding the lack of effectiveness of condoms reported in reducing HPV transmission [49, 50].

## Conclusions

Epidemiological considerations will be needed to determine the best screening approach for different geographical settings within Kenya. A screening interval for HPV 16, HPV 53, and HPV 18, the most prevalent HPV genotypes observed, triaged by age may not be optimal in this study population of HIV- infected women as these genotypes are often found in presence of other pHR/HR genotypes. The concomitant WHO recommendation to initiate HAART when asymptomatic at CD4 count ≥350 cells/μl, instead of at CD4 count ≥200 cells/μl, may offer women the possibility to ensure continuous cervical screening during regular follow up, thereby preventing HPV 16 and HPV 18 induced cervical dysplasia.

Given the high prevalence of HPV 53 in a HIV infected population with abnormal cytology, its cervical carcinoma genesis potential as a stand alone genotype and as well as its synergism with multiple infections should be investigated.

The new WHO guideline to rescreen within ten years may be more effective if it is interspersed with BV management. Given the high prevalence of cervicitis in this population and its potential public health impact, it is important that bacterial ecology monitoring becomes an integral component of HIV-HPV management. To this effect, there is a need for training of health care personnel in validated point of care diagnostics for diagnosing BV in HIV infected women. In addition, it is pivotal that the aetiology of cervicitis be elucidated within this study population and a symptomatic management algorithm for cervicitis be established.

Furthermore, the association between BV and HPV 16 and its related genotypes must be further explored. A high BV prevalence, together with high concomitant STIs detected in our study underscores the need that the possible synergistic effects of co-infections be elucidated for public health interest. In addition, both high concomitant STIs prevalence and very irregular use of condoms make the case for strengthening STD counseling within HIV care.
